# Covalent Grafting of Functionalized MEW Fibers to Silk Fibroin Hydrogels to Obtain Reinforced Tissue Engineered Constructs

**DOI:** 10.1021/acs.biomac.3c01147

**Published:** 2024-02-07

**Authors:** Martina Viola, Madison J. Ainsworth, Marko Mihajlovic, Gerardo Cedillo-Servin, Mies J. van Steenbergen, Mattie van Rijen, Mylène de Ruijter, Miguel Castilho, Jos Malda, Tina Vermonden

**Affiliations:** † Department of Pharmaceutical Sciences, Division of Pharmaceutics, Utrecht Institute for Pharmaceutical Sciences (UIPS), Utrecht University, 3508 TB Utrecht, The Netherlands; ‡ Department of Orthopedics, University Medical Centre Utrecht, 3584 CT Utrecht, The Netherlands; § Department of Biomedical Engineering, Technical University of Eindhoven, 5612 AE Eindhoven, The Netherlands; ∥ Institute for Complex Molecular Systems, Eindhoven University of Technology, 5600 MB Eindhoven, The Netherlands; ⊥ Department Clinical Sciences, Faculty of Veterinary Medicine, Utrecht University, 3584 CS Utrecht, The Netherlands

## Abstract

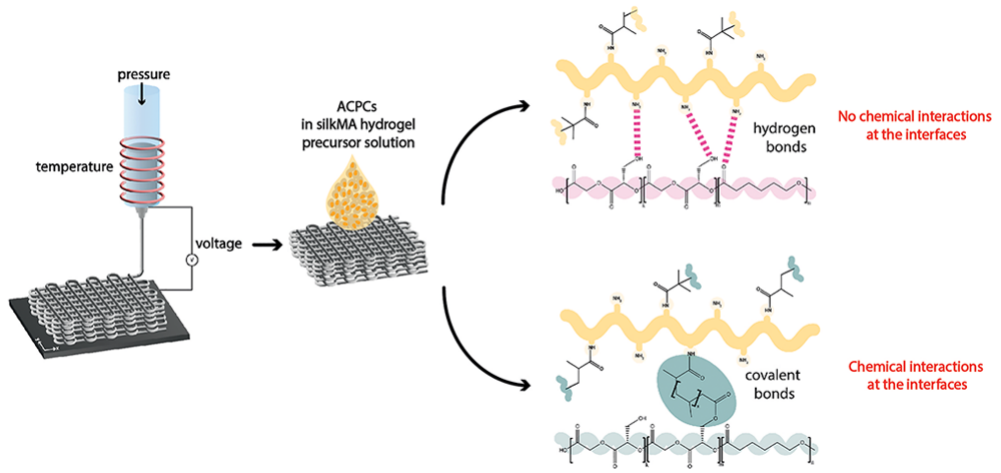

Hydrogels are ideal materials to encapsulate cells, making them suitable for applications in tissue engineering and regenerative medicine. However, they generally do not possess adequate mechanical strength to functionally replace human tissues, and therefore they often need to be combined with reinforcing structures. While the interaction at the interface between the hydrogel and reinforcing structure is imperative for mechanical function and subsequent biological performance, this interaction is often overlooked. Melt electrowriting enables the production of reinforcing microscale fibers that can be effectively integrated with hydrogels. Yet, studies on the interaction between these micrometer scale fibers and hydrogels are limited. Here, we explored the influence of covalent interfacial interactions between reinforcing structures and silk fibroin methacryloyl hydrogels (silkMA) on the mechanical properties of the construct and cartilage-specific matrix production *in vitro*. For this, melt electrowritten fibers of a thermoplastic polymer blend (poly(hydroxymethylglycolide-*co*-ε-caprolactone):poly(ε-caprolactone) (pHMGCL:PCL)) were compared to those of the respective methacrylated polymer blend pMHMGCL:PCL as reinforcing structures. Photopolymerization of the methacrylate groups, present in both silkMA and pMHMGCL, was used to generate hybrid materials. Covalent bonding between the pMHMGCL:PCL blend and silkMA hydrogels resulted in an elastic response to the application of torque. In addition, an improved resistance was observed to compression (∼3-fold) and traction (∼40–55%) by the scaffolds with covalent links at the interface compared to those without these interactions. Biologically, both types of scaffolds (pHMGCL:PCL and pMHMGCL:PCL) showed similar levels of viability and metabolic activity, also compared to frequently used PCL. Moreover, articular cartilage progenitor cells embedded within the reinforced silkMA hydrogel were able to form a cartilage-like matrix after 28 days of *in vitro* culture. This study shows that hybrid cartilage constructs can be engineered with tunable mechanical properties by grafting silkMA hydrogels covalently to pMHMGCL:PCL blend microfibers at the interface.

## 1 Introduction

Hydrogels are defined as hydrophilic three-dimensional polymeric networks able to absorb large amounts of water (up to 90–99% of their volume)^1−4^ and, as such, are often used in tissue engineering and regenerative medicine (TERM) as analogues to the extracellular matrix of natural tissues.^5,6^ Many TERM concepts are based on the assumption that once cells are organized within a 3D environment (generally, in part, provided by hydrogels), cell–cell and cell–material interactions can be induced, which in turn stimulates tissue maturation.^7−9^

Despite the attractive characteristics of many hydrogels, their limited mechanical strength^10−12^ leads to failure under harsh mechanical conditions, as for instance experienced in bone and cartilage tissues when subjected to high pressure^13^ or in the case of blood vessels when subjected to strong shear forces.^14,15^ Growth and differentiation of embedded cells is typically initiated in a soft material environment created by low-density polymer networks.^16,17^ While the simplest approach to directly improve the stiffness and strength of a hydrogel is to increase the polymer concentration and cross-linking density,^18,19^ this is at the expense of the diffusion rate of bioactive factors, nutrients, and cellular metabolites through the matrix.^2,19−21^ Alternative strategies to increase the mechanical strength of hydrogels, without increasing polymer density/concentration,^22^ involve the incorporation of solid particles^23−25^ or nanofibers/nanotubes.^26−30^ However, the incorporation of fillers may introduce challenges related to biocompatibility and potential toxicity when they interact with biological systems. Moreover, achieving the desired mechanical properties while maintaining optimal biocompatibility requires a good balance between fillers and hydrogel concentrations to avoid compromising crucial aspects of the hydrogel’s performance.^26−30^

Another common approach to reinforce soft hydrogel structures is to combine them within three-dimensional (3D) porous scaffolds. Such 3D support structures can be simultaneously printed with the hydrogel material, using techniques based on extrusion^22,31−33^ or inkjet.^34,35^ A novel technique to create 3D fiber reinforced scaffolds is melt electrowriting (MEW).^36^ MEW allows for the generation of micrometer scale fibers that mechanically reinforce hydrogels while only encompassing a low volume percentage of the eventual construct.^37^ MEW offers high fiber resolution with fiber diameters between 0.82–45 μm^38−40^ and scaffold porosities of more than 87%, allowing the cells to be in direct contact with soft materials while bulk mechanical stability is ensured.^38,41^ The use of such scaffolds as reinforcement, resembling the fibrous organization present in the ECM microenvironment, results in a large surface area which favors scaffold–hydrogel interaction, with a majority of the construct volume available for cells as stimulative environment.^38,42−44^ Furthermore, it was shown that highly organized melt electrowritten fibers are mechanically stable enough to provide the challenging loading conditions in for example the equine joint.^45^

Poly(hydroxymethylglycolide-*co*-ε-caprolactone) (pHMGCL), a thermoplastic copolymer of polycaprolactone (PCL), has been used to produce MEW scaffolds in the cardiac tissue engineering field, which was found to be beneficial for cellular attachment and alignment in comparison to pure PCL.^46^ However, including reinforcing scaffold structures to hydrogels often results in biphasic systems due to their different physicochemical properties,^47−50^ leading to inhomogeneity in the mechanical properties of the composite structure.^51^ The importance of the interaction between the different types of materials in composite scaffolds at the interfaces is often overlooked, even though it is known that these interfaces play an important role in the mechanical and biological performance.^51,52^

**Figure 1:**
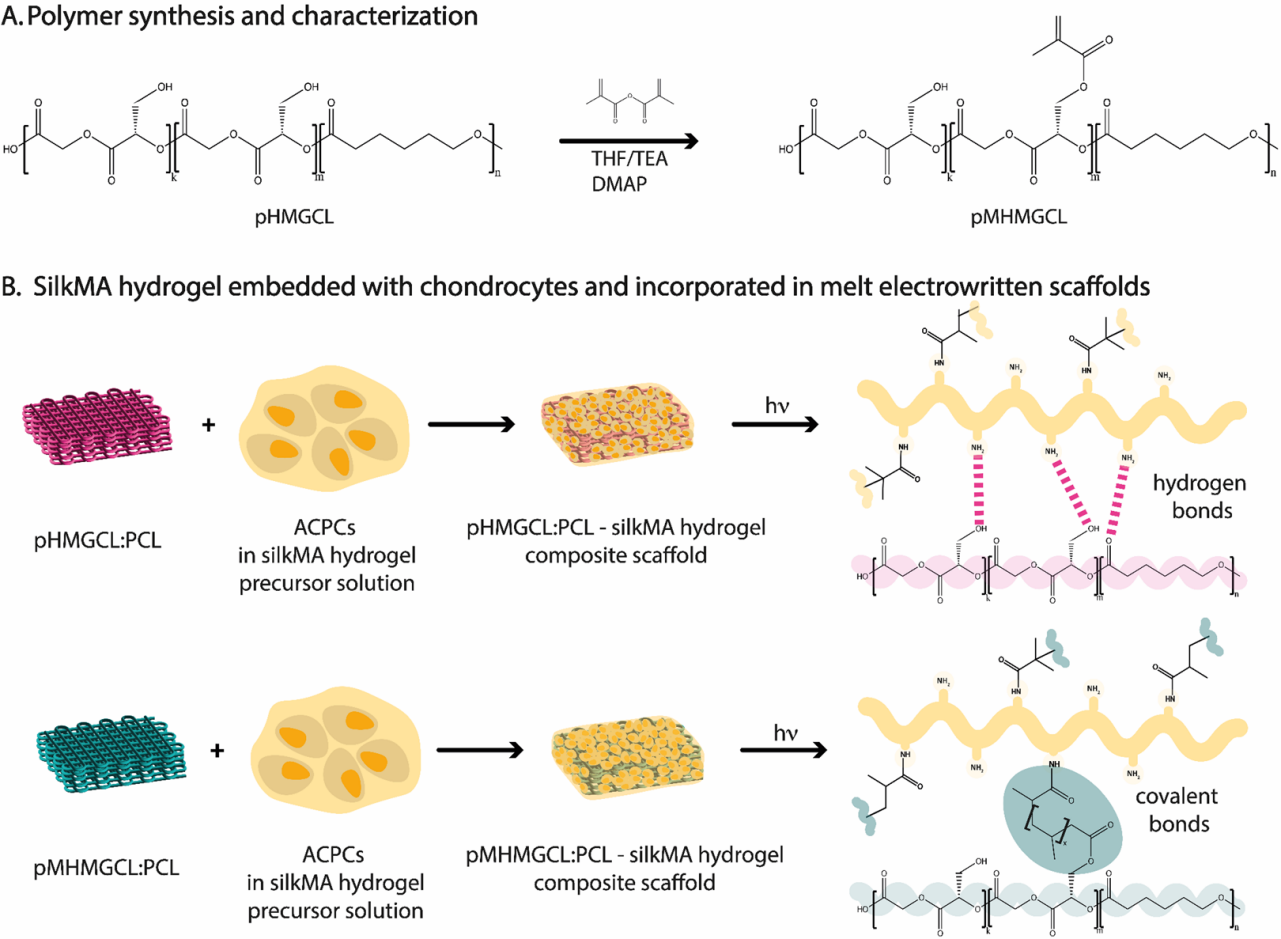
Study rationale: (A) pHMGCL methacrylation reaction; (B) overall process to evaluate the effect of interface interactions on differentiation of equine articular cartilage progenitor cells (ACPCs).

In this study, we aim to tune the mechanical properties of methacrylated silk-fibroin-based hydrogel structures with melt electrowritten reinforcing scaffolds making use of covalent bonds at the interface. The MEW scaffolds are based on pHMGCL functionalized with methacrylic groups linked to the lateral hydroxyl groups (pMHMGCL) (Figure 1).^53^

Previous work demonstrated that pHMGCL was more hydrophilic than PCL due to the presence of hydroxyl side groups, promoting interaction with the hydrophilic matrix of the hydrogels as well as enhancing cell adhesion.^53,54^ Moreover, pHMGCL has also been used in the form of a 3D scaffold obtained through fused deposition modeling (FDM) and was shown to enhance metabolic activity of seeded MSCs when compared to PCL.^54^ The methacrylated polyester was subsequently developed for the fabrication of a reinforced FDM-printed scaffold for cartilage tissue engineering. It was found that the binding strength between the pMHMGCL:PCL and a gelatin methacryloyl hydrogel was over 5 times higher than without the methacrylate functionalization and consequently exhibited enhanced mechanical integrity.^55^ Applying this concept to MEW would allow for finer control of scaffold design, creating ultrafine fibers that closer mimic the natural extracellular matrix structure. This level of precision offers superior control over crucial factors such as cellular attachment, proliferation, and tissue integration. The fine fibers produced by MEW provide the reinforced scaffolds with enhanced mechanical properties compared to those of the hydrogel alone, including increased strength, flexibility, and elasticity. These improvements render the scaffolds more resilient, making them ideal for demanding load-bearing applications.^56−58^ Moreover, incorporating a three-dimensional scaffold into a soft hydrogel allows ideal conditions for cell proliferation of hydrogels combined with mechanical support.

This study investigated the role of the interconnection between the reinforcing fibers and the soft hydrogel component by comparing the presence/absence of covalent bonds at the interface between silkMA hydrogel and p(M)HMGCL:PCL following photo-cross-linking. SilkMA hydrogels reinforced with thermoplastic polymer blends pHMGCL:PCL and pMHMGCL:PCL were compared under uniaxial tensile (and compression) loading conditions. Finally, the *in vitro* cartilage-like matrix production of articular cartilage progenitor cells (ACPCs)^59^ within the constructs reinforced with melt electrowritten pHMGCL:PCL and pMHMGCL:PCL fibers was investigated.

## 2 Materials and Methods

### 2.1 Materials

Lithium phenyl 2,4,6-trimethylbenzoyl phosphinate (LAP) was purchased from TCI Chemicals. *Bombyx mori* cocoons were purchased from Evrosilk. PCL (Purasorb PC12) was purchased from Corbion. Phosphate-buffered saline (PBS) 1x (pH ∼ 7.4) was purchased from Sigma-Aldrich. All other reagents and solvents were purchased from Sigma-Aldrich and used without purification unless stated otherwise.

### 2.2 Silk Fibroin Extraction, Methacrylation (of SF), and Hydrogel Preparation

Silk degumming was based on a protocol by Rockwood et al.,^60^ and silk methacrylation was performed according a protocol by Kim et al.^61^ In short, *Bombyx mori* silk cocoons were cut using scissors. Cocoons (20 g) were boiled in a 0.02 M sodium carbonate (Na_2_CO_3_) aqueous solution (8 L) for exactly 30 min. Silk fibroin (SF) was rinsed in cold deionized water and washed three times in cold deionized water (50 mL) for 20 min. SF was squeezed by hand to remove excess water and dried on aluminum foil overnight. A SF aqueous solution was prepared by dissolving 5 g of dried SF in 9.3 M LiBr solution at 60 °C. After 1 h, glycidyl methacrylate (GMA) (1.12 g, 7.9 mmol) was added, and the solution was left to stir for exactly 3 h. Silk fibroin methacryloyl (silkMA) was purified by dialysis against deionized water for 3 days using cellulose dialysis tubes (MWCO 3.5 kDa, Sigma-Aldrich) at 4 °C and characterized with ^1^H NMR (Figure S1).^61^ The dialyzed solution was centrifuged to remove any further impurities and freeze-dried. SilkMA hydrogel was obtained by dissolving 7% (w v^–1^) silkMA and 0.1% (w v^–1^) LAP in PBS at room temperature; the cross-linking was triggered by UV light for 5 min (Cl-1000, ultraviolet cross-linker, λ = 365 nm, *I* = 8 mW cm^–2^ UVP).

### 2.3 SF Hydrogel Swelling and Mass Loss

Mass loss and swelling studies were performed to study the stability of the hydrogel overtime (Figure S4). SilkMA hydrogels were prepared as previously reported in Section 2.2. All samples (18 in total) were weighted immediately after cross-linking for the initial wet mass (*m*_wet;*t*=0_), and three samples were lyophilized to obtain their dry weights (*m*_dry;*t*=0_). The actual macromer fraction was calculated based on eq 1
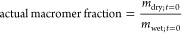
1The remaining samples were then immersed in PBS and incubated at 37 °C. Three samples at the time were removed from the incubator after 1, 3, 7, 14, and 28 days. The excess of water was gently removed and their wet mass was measured (*m*_wet;*t*=*x*_, where *x* represents the day). The swollen samples were then lyophilized to obtain their dry weight (*m*_dry;*t*=*x*_). The swelling ratio was calculated from eq 2, and the mass loss, which represents the degradation of the sample, was calculated from eq 3.
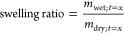
2
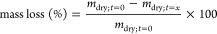
3

### 2.4 Polymer Synthesis

#### Synthesis of Random Copolymer of ε-Caprolactone (CL) and Benzyloxymethyl Glycolide (BMG)^62^ (Poly(benzyloxymethyl glycolide-*co*-ε-caprolactone), pBMGCL)

The polymerization was performed based on a protocol previously reported.^53^ Briefly, CL and BMG were introduced into a dry Schlenk tube equipped with a magnetic stirrer under a dry nitrogen atmosphere in a 3:2 ratio with benzyl alcohol (BnOH) and tin(II) 2-ethylhexanoate (SnOct_2_) as initiator and catalyst, respectively, with a monomer (BMG): initiator ratio of 300:1 and monomer (BMG): catalyst ratio of 300:0.5. The tube was evacuated with nitrogen flow for 2 h at room temperature and then immersed in an oil bath at 130 °C overnight. The polymer was dissolved in chloroform, precipitated in cold methanol three times, and dried under vacuum overnight. The obtained polymer was characterized with proton nuclear magnetic resonance (^1^H NMR), gel permeation chromatography (GPC) and differential scanning calorimetry (DSC).

^1^H NMR (CDCl_3_): δ 1.3–1.4 (m, CH_2_–CH_2_–C*H*_2_–CH_2_–CH_2_), 1.5–1.7 (m, CH_2_–CH_2_–CH_2_–C*H*_2_–CH_2_), 2.3 (t, CH_2_–C*H*_2_–CO), 2.4 (t, CH_2_–C*H*_2_–CO), 3.7–4.0 (m, CH–C*H*_2_), 4.0 (t, O–C*H*_2_–CH_2_), 4.0 (t, O–C*H*_2_–CH_2_), 4.4–4.9 (m, C*H*_2_–Ar, O–C*H*_2_–CO), 5.1–5.5 (m, C*H*), 7.2–7.4 (m, C–*H*Ar).

#### Removal of Benzyl Protecting Groups

Protecting benzyl groups of pBMGCL were removed in a hydrogenation reaction using palladium on carbon (Pd:C) catalyst as previously reported.^53^ Briefly, pBMGCL (1 g) was dissolved in dry tetrahydrofuran (THF, 30 mL) with Pd:C (50 mg, 0.12 mmol) in a dry round-bottom flask. The flask was filled with hydrogen in three consecutive steps of evacuation and refilling with H_2_, and the reaction was done under hydrogen (H_2_) pressure overnight at room temperature. The catalyst was removed with two centrifugation steps followed by filtering over Celite. THF was removed by evaporation. The polymer was characterized by ^1^H NMR, GPC, and DSC.

^1^H NMR (CDCl_3_): δ 1.2–1.4 (m, CH_2_–CH_2_–C*H*_2_–CH_2_–CH_2_), 1.5–1.7 (m, CH_2_–C*H*_2_–CH_2_–CH_2_–CH_2_, O–CH_2_–C*H*_2_–C*H*_2_–CH_2_–O), 2.3 (t, CH_2_–C*H*_2_-CO), 2.4 (t, CH_2_–C*H*_2_–CO), 3.7–4.4 (m, CH–C*H*_2_, O–C*H*_2_–CH_2_), 4.5–5.1 (m, O–C*H*_2_–CO), 5.0–5.4 (m, C*H*).

#### Partial Methacrylation of Hydroxyl Groups to Prepare Methacrylated pHMGCL (pMHMGCL)

pHMGCL (500 mg) was dissolved in THF (5 mL) in an aluminum foil covered, dry round-bottom flask. After dissolution, 4-(dimethylamino)pyridine (DMAP; 5 mg, 0.04 mmol) and triethylamine (107 μL, 0.71 mmol) were added as catalyst and base, respectively. Then, methacrylic anhydride (115 μL, 0.77 mmol; feed ratio methacrylic anhydride:OH groups on polymer = 0.5) was added. To prevent premature cross-linking, hydroquinone monomethyl ether (10 mg, 0.08 mmol) was added. The reaction proceeded overnight under nitrogen (N_2_) in an ice-cooled flask. The polymer was purified by three times precipitation in ice-cold water, followed by centrifugation and removal of supernatant. The precipitate was redissolved in dichloromethane (DCM) and dried using anhydrous sodium sulfate (Na_2_SO_4_). The salts were filtered off, DCM was evaporated, and the polymer was dried under vacuum at room temperature overnight. The polymer was characterized by ^1^H NMR, GPC, and DSC.

^1^H NMR (CDCl_3_): δ 1.2–1.4 (m, CH_2_–CH_2_–C*H*_2_–CH_2_–CH_2_), 1.5–1.7 (m, CH_2_–C*H*_2_–CH_2_–CH_2_–CH_2_, O–CH_2_–C*H*_2_–C*H*_2_–CH_2_–O), 1.9–2 (m, C*H*_3_–C−), 2.3 (t, CH_2_–C*H*_2_–CO), 2.4 (t, CH_2_–C*H*_2_–CO), 3.7–4.4 (m, CH–C*H*_2_, O–C*H*_2_–CH_2_), 4.5–5.1 (m, O–C*H*_2_–CO), 5.0–5.4 (m, C*H*), 5.6–6.4 (t, C*H*_2_=C−).

#### Preparation of 1:1 Blend pHMGCL:PCL and 1:1 Blend pMHMGCL:PCL

pHMGCL or pMHMGCL (200 mg) was dissolved in a 1:1 ratio with PCL in DCM (5 mL). The mixture was left to dry overnight in a Petri dish in a fume hood. The polymer blend was analyzed with DSC and thermogravimetric analysis (TGA).

### 2.5 ^1^H NMR Spectroscopy

^1^H NMR spectra were recorded using an Agilent Technologies 400 MHz MR spectrometer. The samples were prepared by mixing ∼5 mg of each sample in 800 μL of deuterated chloroform (CDCl_3_). Chemical shifts are recorded in parts per million with reference to the solvent peak (δ 7.26 ppm for CDCl_3_).

### 2.6 Gel Permeation Chromatography (GPC)

GPC for polymer analysis was performed using an Alliance 2695 (Waters) chromatography system with a MIXED-D column (Agilent PLgel) and equipped with a Waters 2489 UV/vis detector and a Waters 2414 refractive index detector. The method was calibrated against polystyrene standards of known *M*_w_ from EasiCal PS-2 (PL2010-0601, PL2010-0605). Chloroform was used as a mobile phase with an elution flow rate of 1 mL min^–1^ at 30 °C. Sample concentration was 5 mg mL^–1^. Recording of data and calculations of molecular weights (*M*_w_) were done using Waters Empower 32 software.

### 2.7 Differential Scanning Calorimetry (DSC)

Thermal properties of the polymers and the blends were measured using a DSC Q2000 instrument (TA Instruments). A cycle of scans (heating–cooling–heating) was performed on polymer samples (5 mg, loaded into Tzero aluminum pans (TA Instruments)) from 0 to 200 °C at a heating rate of 10 °C min^–1^ and a cooling rate of 1 °C min^–1^ under a nitrogen flow of 50 mL min^–1^. Melting temperatures (*T*_m_) were determined from the onset of the endothermic peaks of the second heating run.

### 2.8 Thermogravimetric Analysis (TGA)

The degradation temperature was determined with a TGA Q500 (TA Instruments). Temperature ramps up to 150 °C with a 10 °C min^–1^ rate were measured for all polymer blends (∼10 mg loaded into platinum pans). Degradation times were determined by temperature ramps up to 80 °C with a 10 °C min^–1^ rate, followed by an isothermal scan at 80 °C for 24 h.

### 2.9 Static Contact Angle Measurements

Changes in polymer blends’ surface wettability were evaluated by static contact angle measurements using the sessile drop technique (Data Physics, OCA 15EC). All measurements (*n* = 3) were performed on uniform polymer films of each composition with a water droplet of 10 μL and repeated in triplicate. Contact angles were measured by averaging the right and left angles of the water droplet by using the surface contact angle software (SCA20, Data Physics).

### 2.10 Creep-Recovery Test

To show the effect of the presence/absence of covalent bonds between silkMA hydrogel and the two polymer blends pHMGCL:PCL and pMHMGCL:PCL, creep-recovery tests were performed with a rheometer (Discovery HR2, TA Instruments), equipped with a fitted EHP upper plate and with a light guide attached to a BluePoint 4 lamp (Honle UV technology) at 37 °C, using a 20 mm plate–plate geometry. pHMGCL (or pMHMGCL) and PCL were dissolved in a 1:1 ratio in 10 mL of DCM. The solution was poured in a 94 mm diameter, 16 mm height Petri dish and left dry overnight. Flat discs (surface area 28.3 mm^2^, thickness 0.1 mm) of pHMGCL:PCL and pMHMGCL:PCL were prepared by punching the film using biopsy punches (diameter 6 mm). The silkMA solution was prepared at a concentration of 7% w v^–1^ in PBS with 0.1% w v^-1^ LAP. The pHMGCL:PCL or pMHMGCL:PCL film was attached with a photoadhesive sticker (HEMA) to the top plate of the rheometer, and then 70 μL of silkMA solution (7% w v^–1^ + 0.1% w v^–1^ LAP) was pipetted onto the bottom plate. The gap between the two plates (so between the polymer film and the silkMA hydrogel) was set at 1 mm. The interface was irradiated with UV light for 5 min (λ = 365 nm, *I* = 8 mW cm^–2^ UVP). A constant stress was applied to the interface (5, 10, or 20 Pa) for 5 min, followed by a 5 min recovery for 10 cycles, maintaining a constant temperature of 37 °C. The deformation of the interface was recorded in the creep step (when stress was applied); then, once the force was released, the recovery, if any, of the material was recorded. For each polymer blend, measurements were performed in triplicate (*n* = 3) and reported as the average value.

### 2.11 Melt Electrowriting of Polymer Blends

MEW was performed using an in-house set up for scaffold manufacturing as described previously.^63^ The polymer blend (pHMGCL:PCL or pMHMGCL:PCL) was placed in a 3 mL glass syringe (Fortuna Optima Ganzglasspritze, Poulten & Graf GmbH) with a 27G metal needle (Unimed) connected to a sealed hose delivering pressurized nitrogen (VPPE-3-1-1/8-2-010-E1, Festo). The polymer blend was heated at 80 °C using a heating module composed of an electrical heating coil element wrapped around the glass syringe and directly connected to a temperature regulator (TR 400, HKEtec). The polymer blend was electrified using a high voltage source (Heinzinger, LNC 10000-2neg) and collected onto a grounded collector plate (*x*–*y*), controlled by an advanced motion controller Motion Perfect v5.0.2 (Trio Motion Technology Ltd.).

Polymer processing was optimized according to key MEW parameters, specifically the voltage (*V*), the pressure (*p*), the collector speed (CS), the collection distance (CD), and temperature (*T*). Fiber diameter and morphology were investigated with microscopy, and the mentioned parameters were changed one parameter at a time for the following value ranges: *V* = 5–7 kV, *p* = 0.5–2 bar, CS = 50–600 mm s^–1^, CD = 2–6 mm, and *T* = 80 °C. Several (at least 10) single fibers were printed, at each parameter combination, on glass slides and examined using a polarized light microscope (BX51P, Olympus). The number of fibers used for the diameter measurements was at least 10. The same optimized parameters were used for both blends. Well-organized squared scaffold meshes (40 × 40 mm^2^) were programmed and fabricated with a fiber-to-fiber spacing of 400 μm in a square architecture and 300 stacked layers, where 1 layer was defined as a line pattern in *x* + *a* line movement in the *y*-direction to achieve the box-shaped pattern.

PCL scaffolds were produced on a bioprinting system (3DDiscovery, RegenHU), and the parameters were optimized: *T* = 75 °C, *V* = 7.44 kV, *p* = 1.3 bar, CS = 8.5 mm s^–1^, and CD = 4 mm. Well-organized squared scaffold meshes (52 × 52 mm^2^) were programmed and fabricated with a fiber-to-fiber spacing of 400 μm and 32 stacked layers (defined as above; to match final scaffold thickness between polymers used in this study, we accounted for the difference in fiber diameter of polymer blend).

Two different printers were used for the polymer blends, and PCL was used to enable printing at different speeds. As can be seen from the printing parameters, the polymer blends need a high printing speed of 300 mm s^–1^, while PCL printing was performed at a lower speed.

Printed scaffolds were visualized using a SEM Phenom Pro (Thermo Fisher Scientific). Prior to scanning, circular samples with a diameter of 3 mm were cut from the MEW meshes and sputter-coated with an 8.3 nm Pt:Pd layer using a sputter coater 208HRD with a rotary-planetary-tilt stage (Cressington). Using the scaffold’s SEM images, the fiber diameter, fiber spacing, and quality number (this value varies between 0 and 1, where 0 indicates no stacking and 1 indicates perfect stacking) were measured using ImageJ software (version 2.9.0/1.5t).

### 2.12 Sample Preparations

Samples were prepared by combining a silk precursor solution with a polymer mesh. For all experiments that do not contain cells, sample preparation was performed as follows: squared meshes (40 × 40 mm), with fiber-to-fiber spacing 400 μm and 300 stacked layers, were casted with 1.2 mL of 7% (w v^–1^) silkMA and 0.1% LAP in PBS and exposed to UV light for 5 min (Cl-1000, ultraviolet cross-linker, λ = 365 nm, *I* = 8 mW cm^–2^ UVP). The volume of silkMA solution was measured in relation to the scaffold volume (1200 mm^3^) [the precise thickness of each scaffold (∼0.75 mm) was measured using a height gauge and multiplied by the area of the square (40 × 40 mm^2^ = 1600 mm^2^)]. The casting was done inside a 40 mm × 40 mm mold to ensure a homogeneous solution distribution.

The preparation of samples containing cells was performed as follows: PCL scaffold sheets were etched in a 1 M sodium hydroxide (NaOH) solution for 30 min. Repeat Milli-Q water rinses were performed until the pH reached 7. pHMGCL:PCL and pMHMGCL:PCL scaffolds were not etched prior to culture *in vitro*. Squared meshes (40 × 40) were cut using a 5 mm ϕ biopsy punch and sterilized by 30 min of submersion in 70% ethanol, followed by UV irradiation (254 nm wavelength) for 20 min per side. Scaffolds were stored in ACPCs expansion medium at 4 °C overnight prior to seeding procedure. Two mL of 7% (w v^–1^) silkMA solution with a 0.1% (w v^–1^) LAP was filtered (0.22 μm), and 40 × 10^6^ cells were suspended in it. 30 μL of silkMA with ACPC suspended cells (∼5 × 10^5^ per sample) were seeded onto each scaffold with expansion medium (-bFGF) and left to attach for 6 h in suspension well plates, adding media as needed to keep samples moist.

### 2.13 Uniaxial Tensile Tests

pHMGCL:PCL and pMHMGCL:PCL scaffolds were prepared as explained in Section 2.11. From every cast scaffold, 6 samples were obtained by cutting it in equal parts (10 mm × 20 mm) with a surgical bistoury. Uniaxial tensile tests were performed using a BioTester 5000 device (CellScale) and a 5 N load cell in PBS (1x). pHMGCL:PCL (or pMHMGCL:PCL)-silkMA reinforced samples were tested under quasi-static monotonic conditions at a strain rate of 20% min^–1^ (*n* = 6). Force–displacement curves were recorded by using LabJoy software (CellScale) and normalized to obtain engineering stress–strain curves. Tension modulus values were calculated from least-squares fitting of the slope in the linear region of the stress–strain curves. Moreover, the breaking point stress and strain were calculated and quantified. The same test was performed on pHMGCL:PCL and pMHMGCL:PCL scaffolds without hydrogel to test the mechanical properties of the fibers alone.

### 2.14 Cell Expansion

Equine ACPCs were isolated as previously described^59^ according to the medical ethics regulations of the University Medical Center Utrecht and the guideline “good use of redundant tissue for research” of the Dutch Federation of Medical Research Societies.^64^ After isolating, ACPCs (seeding density ≈7.73 × 10^3^ cm^–2^) were stimulated to proliferate on conventional tissue culture plastic in ACPC expansion medium; Dulbecco’s modified Eagle’s medium (DMEM; Gibco), 10% heat-inactivated fetal bovine serum (VWR), 1% penicillin/streptomycin (100 U mL^–1^; Gibco), 200 μM 1-ascorbic acid 2-phosphate (Sigma-Aldrich), 1X nonessential amino acids (Gibco), 5 ng mL^–1^ basic fibroblast growth factor (PeproTech). Media was refreshed twice per week until ∼80% confluency was reached at passage 5.

### 2.15 *In Vitro* Experiments

#### 2.15.1 Viability and Metabolic Activity Test

PCL, pHMGCL:PCL, and pMHMGCL:PCL scaffolds were prepared as explained in Section 2.12. After attachment was microscopically observed, the ACPC expansion medium (-bFGF) was added to scaffolds. Scaffolds were kept in culture for 1 week, with time points for the Live/Dead assay on D1 and D7 (*n* = 2), and time points for the metabolic assay on D1, D4, and D7 (*n* = 4). Cell-free scaffolds were taken along in culture for each of the assays to measure background fluorescence. Remaining samples on D4 were transferred to new suspension well plates with a fresh ACPC expansion medium (-bFGF).

##### Live/Dead Assay

2 μM calcein-AM (Invitrogen) was used to stain live cells, and 4 μM ethidium homodimer-1 (Invitrogen) was used to stain dead cells according to the manufacturer’s protocol. Samples were imaged using Leica Thunder microscope using 10× objective with *z*-volume of 200 μm with 5 μm steps and with 20× objective with *z*-volume of 100 μm with 5 μm steps. Images were processed using Leica LAS X software with instant computational clearing with 70% strength.

##### Metabolic Activity Assay

Samples were measured for metabolic activity using a resazurin assay (resazurin sodium salt, Alfa Aesar). Briefly, a working solution was prepared in ACPC expansion medium containing 44.11 μM resazurin sodium salt. Samples were incubated in working solution, protected from light, for 4 h at 37 °C. Fluorescence was measured in duplo with excitation at 544 nm and emission at 590 nm.

#### 2.15.2 Biofunctionality Test: Chondrogenic Differentiation

pHMGCL:PCL and pMHMGCL:PCL scaffolds were prepared using a 6 mm ϕ biopsy punch and sterilized by 30 min of submersion in 70% ethanol, followed by UV irradiation for 20 min/side. Cells were collected and resuspended in filter-sterilized 7% w v^–1^ silkMA with 0.1% LAP (in PBS) to a density of 20 × 10^6^ cells mL^–1^. A cell–gel mix (15 μL) was pipetted into an in-house Teflon mold system (sample ϕ = 6 mm). A scaffold was then transferred to each gel droplet and then cross-linked using UV light for 5 min (Cl-1000, Ultraviolet Cross-linker, λ = 365 nm, *I* = 8 mW cm^–2^ UVP). Following cross-linking, samples were submerged in ACPC chondrogenic medium; DMEM, 1% penicillin/streptomycin (100 U mL^–1^; Gibco), 200 μM 1-ascorbic acid 2-phosphate (Sigma-Aldrich), 1% ITS Premix (Corning), 10 mM HEPES (Gibco), 40 ng mL^–1^ dexamethasone (Sigma-Aldrich), 10 ng mL^–1^ TGFB1 (PeproTech). Scaffolds were kept in culture for 28 days, with time points on D1, D14, and D28 for compressive, biochemical, and matrix production analysis. Cell-free scaffolds were taken along in culture for each of the assays to measure background. Media was changed twice per week.

##### Compressive Analysis

At each time point (D1, D14, D28), compression tests were performed (*n* = 5) on a 2980 DMA (TA Instruments) with a strain ramp 20% min^–1^ up to 30% of compression. The compression modulus was calculated as the initial slope of the stress–strain curve that was obtained from the compression test.

##### Glycosaminoglycan Quantification

Following compressive tests on D1, D14, and D28, samples were collected, frozen at −20 °C, and lyophilized (*n* = 5). Samples were digested using 200 μL of papain buffer, comprising 0.2 M NaH_2_PO_4_ and 0.01 M EDTA·2H_2_O (pH = 6), mixed with 7.75 units mL^–1^ papain solution and 1.57 mg mL^–1^ cysteine HCl. Samples were digested overnight at 60 °C and then assayed for DNA and GAG content using the Picogreen assay kit (Thermo Fisher Scientific) and dimethyl methylene blue assay (DMMB; Sigma), respectively. Briefly, DMMB solution was prepared in-house (pH = 3). Chondroitin sulfate C was used to prepare a standard curve (0–10 μg mL^–1^). Absorbance was measured on a CLARIOstar Plus microplate reader (BMG Labtech) in duplo at 525 and 595 nm; the ratio of the absorbance, 525/595, was taken followed by subtracting the blank.

##### Histological Analysis

At each time point (D1, D14, and D28), samples (*n* = 3 + 1 cell-free sample) were fixed in formalin for 30 min and then stored in PBS at 4 °C until all samples were collected. Samples were prestained for 24 h with 0.1% eosin (in 4% formalin) for general tissue staining to assist with cutting placement. Samples were embedded in 4% agarose and then underwent standard tissue processing and paraffin embedding. Following paraffin embedding, samples were cut to 5 μm thickness and stained with safranin-O (Saf-O) for glycosaminoglycan visualization, fast green for cytoplasm, and Weigert’s hematoxylin for cell nuclei. Immunohistochemical staining of collagen type II was also performed on the paraffin sections as previously described using the primary antibody II-II6B3 (DSHB).^65^ Histology images were made of mounted sections in 10x random locations using a bright-field microscope (BX43; Olympus).

### 2.16 Statistical Analysis

All data are shown as mean ± standard deviation (SD). Statistical significance was tested by unpaired *t* test with Welch’s correction or two-way ANOVA with Tukey’s multiple comparisons test. All statistical analysis was performed with Prism software (GraphPad, version 9.5.1).

## 3 Results and Discussion

### 3.1 Polymer Synthesis and Characterization

Poly(benzyloxymethylglycol-*co*-ε-caprolactone) (pBMGCL) was synthesized through ring-opening polymerization (ROP) of protected benzyl hydroxymethylglycol (BMG) and ε-caprolactone (CL) at a ratio of 3:2 in a melt at 130 °C for 16 h, resulting in the formation of a fine white powder. The obtained monomer ratio as measured using ^1^H NMR spectroscopy yielded a 1:1 ratio. The lower amount of BMG monomer in the polymer chain compared to the feed amount is likely attributed to the error in the integration due to overlapping polymer peaks (Figure S2A).

**Figure 2:**
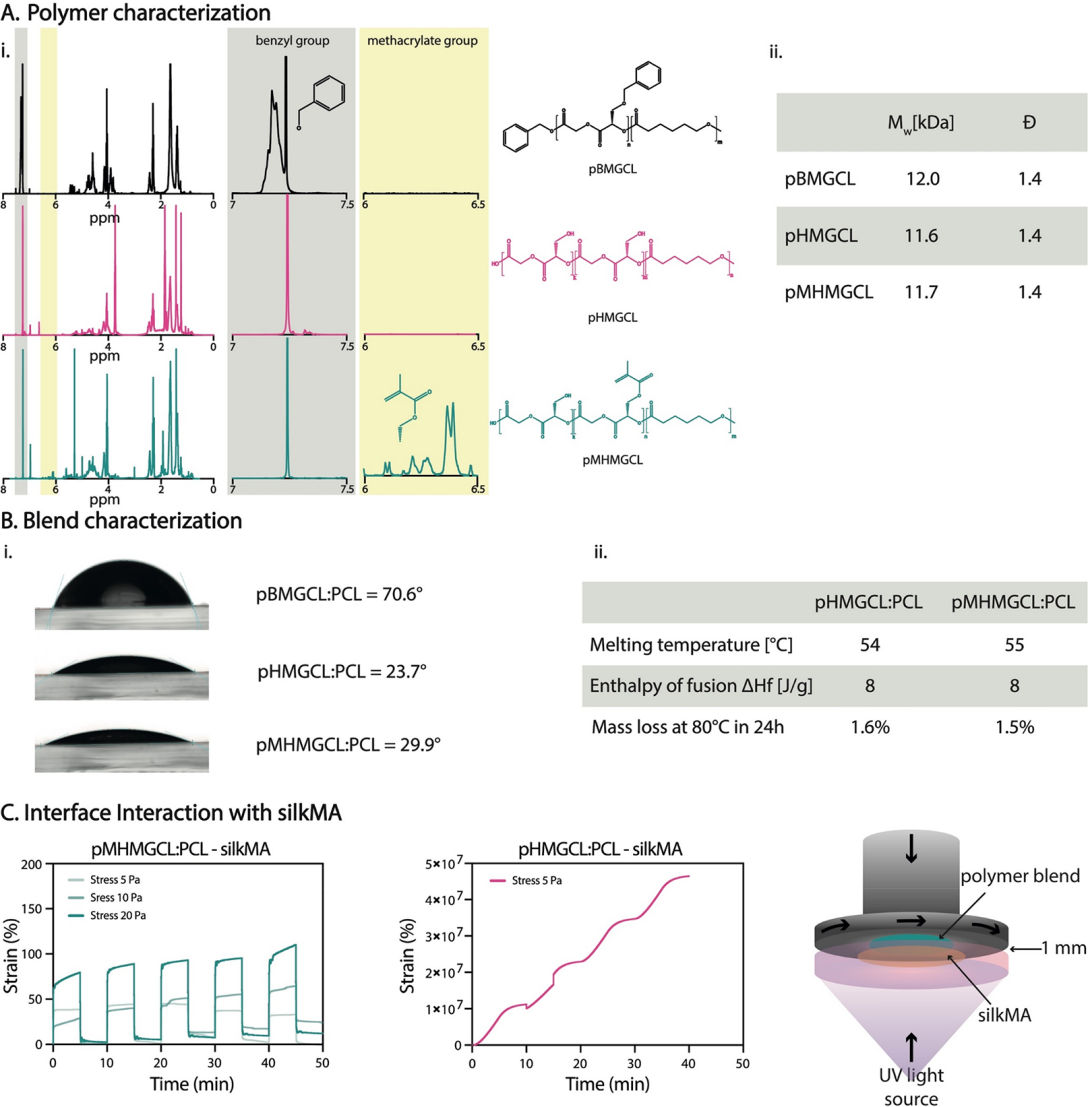
Polymer and polymer blend characterization and assessment of the interface interaction with the silkMA hydrogel. (A) Polymer characterization: (i) ^1^H NMR, (ii) molecular weight (*M*_w_) and polydispersity (*Đ*). (B) (i) Contact angle of the different blends; (ii) characterization of the polymer blends: melting temperature and thermal stability. (C) Creep-recovery experiment (*n* = 3, representative curves are shown).

The benzyl protecting group of pBMGCL was completely removed to obtain pHMGCL, as evidenced by the absence of a signal between 7.0 and 7.5 ppm in the second ^1^H NMR spectrum in Figure 2Ai. Following pHMGCL functionalization, the ^1^H NMR spectrum exhibited characteristic signals of the methacrylic group at approximately 1.9, 5.6, and 6.1 ppm, consistent with previous investigations.^51^ The integration of these signals allowed us to calculate a degree of methacrylation of approximately 20% (Figure S2B).

The molecular weights (*M*_w_) of pBMGCL, pHMGCL, and pMHMGCL were found to be similar, approximately 12.0, 11.6, and 11.7 kDa, respectively, with a polydispersity index (*Đ*) around 1.4 for all polymers. This analysis confirmed that neither the deprotection nor the methacrylation reaction affected the polydispersity of the polymers, consistent with previous reports.^51^ DSC analysis revealed a melting temperature of approximately 47 °C for pBMGCL. However, no endothermic peaks were observed for both pHMGCL and pMHMGCL, indicating that the removal of the protection group alters not only the hydrophilicity but also the molecular mobility of the copolymers (Figure 2Aii). These results align with previous studies, which have shown that an hydroxymethylglycolide (HMG) content above 40% in the polymer chain leads to increased hydrophilicity and inhibits crystallization.^53,55^

Because pHMGCL and pMHMGCL are both amorphous polymers that exist in the form of dense oils at room temperature, they were blended with PCL in a 1:1 ratio to introduce crystallinity and facilitate the preparation of solid scaffolds of sufficient dimensional stability.

The wettability of the polymer blend surfaces was examined for the three blends (pBMGCL:PCL, pHMGCL:PCL, and pMHMGCL:PCL). The removal of the protecting group resulted in increased wettability (Figure 2Bi). However, the introduction of methacrylate groups did not significantly affect the wettability of the blend surfaces. As expected, mixing PCL (with a contact angle of 70°)^46^ with pHMGCL or pMHMGCL led to a reduction of hydrophilicity of the material. Nevertheless, the contact angles of both pHMGCL:PCL and pMHMGCL:PCL blends remained lower than those for PCL alone.

Both pHMGCL:PCL and pMHMGCL:PCL polymer blends exhibited a mass loss of ∼1.5% after 24 h at 80 °C, which corresponds to the temperature used for MEW (Figure 2Bii). This mass loss is considered negligible and is often attributed to the loss of residual solvent traces in the sample during measurement. This result suggests that the polymers remain stable during the printing process without significant degradation. Further investigations into the degradation threshold revealed minimal mass loss, indicating no degradation up to 140 °C (Figure S3).

To investigate the formation of covalent bonds between pMHMGCL:PCL and silkMA hydrogel, rheological creep-recovery measurements were performed on casted polymer blend films (Figure 2C). The pMHMGCL:PCL film silkMA exhibited nearly constant deformation over 5 cycles and nearly 100% recovery upon stress removal for the three applied stress values (5, 10, and 20 Pa). In contrast, the pHMGCL:PCL film–silkMA sample, which was unable to form covalent bonds at the interface, demonstrated an increase in deformation without any observed recovery (Figure 2C).

### 3.2 Investigation of Processing Parameters on Fiber Shape and Diameter

**Figure 3:**
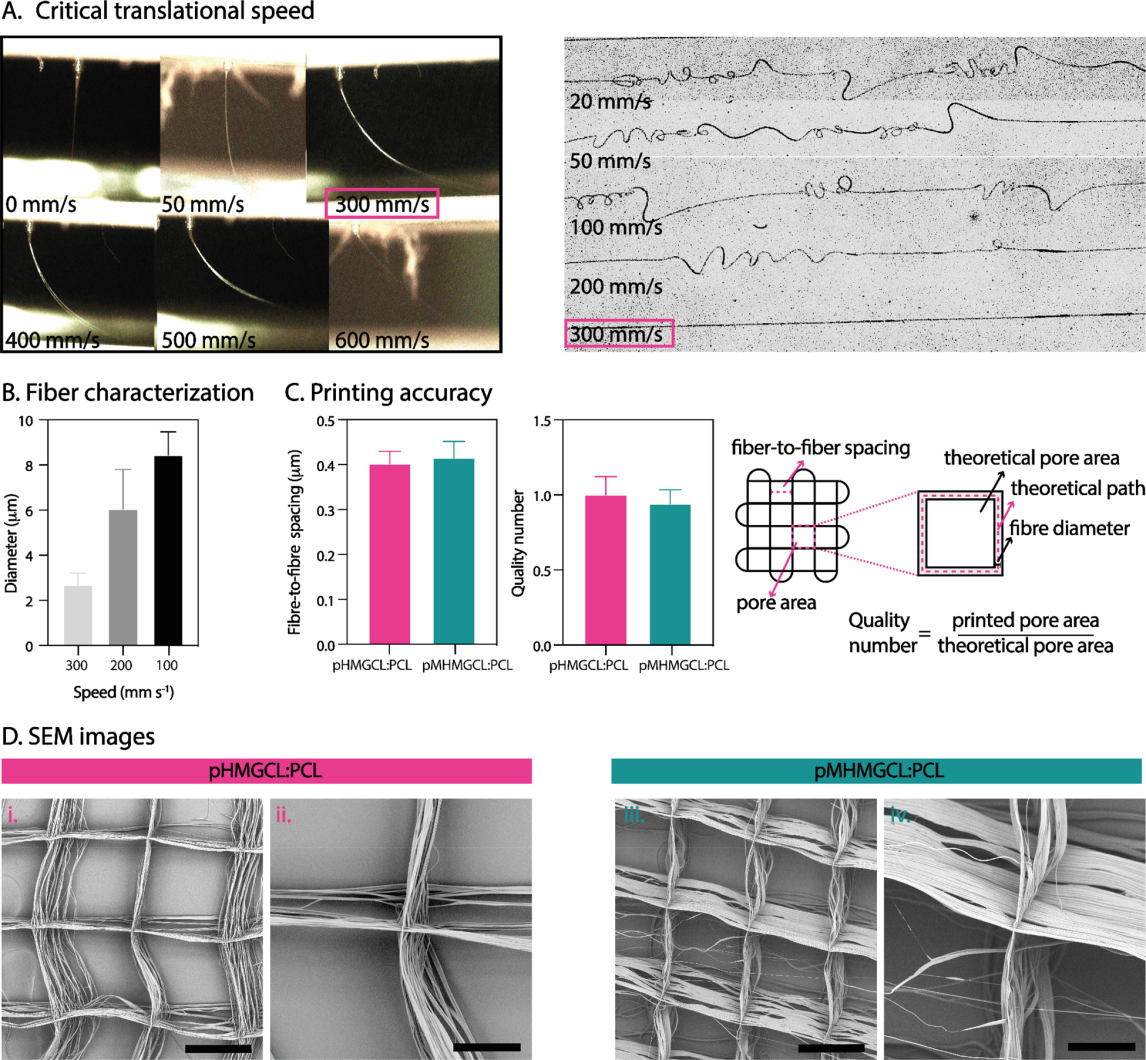
Optimization and accuracy of melt electrowriting polymer blends. (A) Critical translational speed: jet shape and fiber shape. (B) Diameter characterization. (C) Printing parameter evaluation. (D) SEM of pHMGCL:PCL and pMHMGCL:PCL blends scaffolds with 300 layers (scale bar for i and iii = 300 μm and for ii and iv = 100 μm).

Polymer jet formation and the dependence of fiber shape and diameter on printing parameters were investigated for the pHMGCL:PCL and pMHMGCL:PCL formulations (Figure 3A). The same parameters were used for printing both blends: *V* = 5 kV, *p* = 1.2 bar, CS = 300 mm s^–1^, and CD = 4 mm because no significant differences were found in the optimal printing parameters between pHMGCL:PCL and pMHMGCL:PCL with parameters resembling those previously used for PCL and for a similar polymer blend^46^ (*V* = 5 kV, *p* = 1.2 bar, and CD = 4 mm). However, the transition of sinusoidal fibers into straight fibers occurred at higher speed compared to PCL, which showed an average critical translation speed (CTS), the required speed to obtain straight fibers, of about 20–30 mm s^–1^ compared to 300 mm s^–1^ of our blends.^46^ This behavior can be attributed to the lower *M*_w_ of pHMGCL and pMHMGCL compared to PCL, resulting in lower blend viscosities when compared to molten PCL. After determining the CTS for both polymer blends, we examined how the diameter of individual fibers varied with the applied speed. As expected, the diameter decreased from 10 μm to approximately 3 μm as the speed increased from 100 to 300 mm s^–1^ (Figure 3B). For further experiments, the speed was set at 300 mm s^–1^, which guaranteed a fiber diameter of around 3 μm. This diameter was selected based on the notion that cells are more likely to adhere to fibers close to their own size.^66^ However, because thinner fibers can pose challenges in scaffold handling, it was necessary to stack at least 300 layers to obtain a stable scaffold. This aligned with the aim of fabricating a scaffold with increased thickness to resemble the native thickness of articular cartilage.^67^ With regard to the printing temperature, we adhered to the temperature used for PCL of 80 °C,^68^ as both blends were stable over time at this temperature, as confirmed by TGA analysis (Figure 2Bii).

### 3.3 Fiber Scaffold Manufacturing

To ensure the manufacturing of 3D scaffolds with consistent geometry and pore size, the ability to stack microfibers on top of each other was assessed. SEM analyses revealed high accuracy in fiber stacking for square grid geometry for both blends. The analyses were conducted on 300-layer scaffolds with a fiber-to-fiber spacing of 400 μm. Figure 3C shows the uniformity in fiber-to-fiber spacing, both in μm and as a quality number, i.e., the ratio between the measured and theoretical pore area. From Figure 3C, it can be observed that the quality number is very close to 1 for both polymer blends, indicating excellent stacking. However, despite the high-quality number, SEM images (Figure 3D) revealed the presence of some random fibers crossing the rectangular grid geometry. Because the scaffolds are 300 layers in height, the impact of these few random crossing fibers can be neglected.

To summarize, the optimization of MEW process parameters resulted in uniform fiber shape and diameter and successful stacking of microfibers ensured consistent scaffold geometry and pore size.

### 3.4 Mechanical Testing of the MEW Fibrous Scaffold Casted with SilkMA Hydrogel

**Figure 4:**
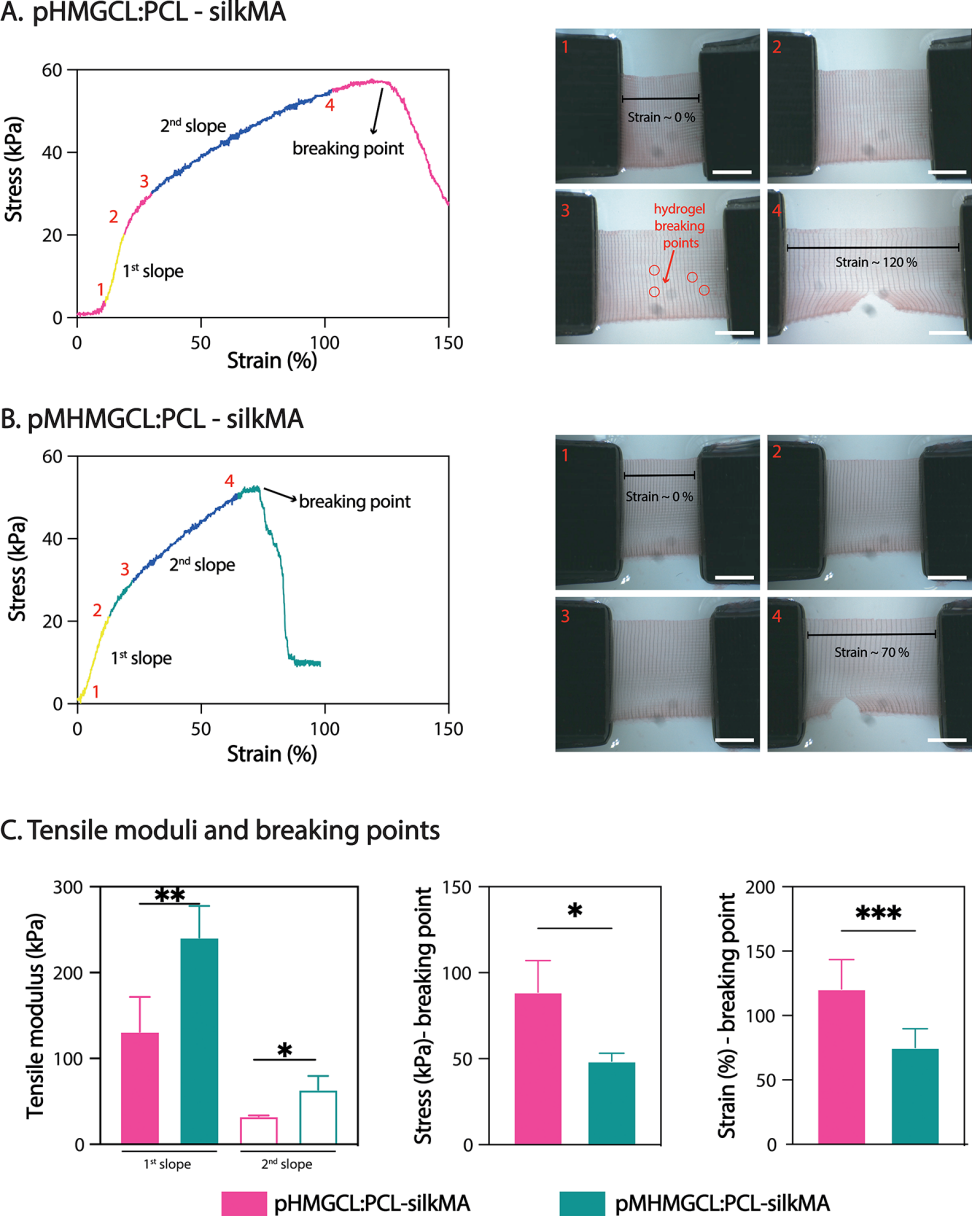
Mechanical characterization polymer blends with silkMA. (A) Representative curves for the uniaxial tensile tests of pHMGCL:PCL-silkMA and top-view photographs of the uniaxial tensile testing setup for pHMGCL:PCL-silkMA: 1, starting point; 2, maximum elastic elongation; 3, plastic (nonelastic) deformation; 4, breaking point (scale bar = 5 mm). (B) Representative curves for the uniaxial tensile tests of pMHMGCL:PCL-silkMA and top-view photographs of the uniaxial tensile testing setup for pMHMGCL:PCL-silkMA: 1, starting point; 2, maximum elastic elongation; 3, plastic (nonelastic) deformation; 4, breaking point (scale bar = 5 mm). (C) Tension modulus calculated on the first and second slope of the curves; values of the stress and the strain at the breaking point for pHMGCL:PCL-silkMA and pMHMGCL:PCL-silkMA scaffolds (*n* = 6). Significance values: *p ≤ 0.05, **p ≤ 0.01, ***p ≤ 0.001.

Mechanical properties were evaluated under uniaxial tensile loading conditions for pHMGCL:PCL and pMHMGCL:PCL scaffolds casted with silkMA hydrogel (7% with 0.1% LAP), revealing nonlinear stress–strain behavior in all cases (Figure 4). The stress–strain curves (Figure 4A,B and Figure S5) exhibited two distinct linear regions (where the first slope is labeled in yellow and the second slope is labeled in blue in Figure 4), with the initial slope being steeper than the subsequent slope for both scaffold groups. Tensile moduli calculated from the angular coefficient of the intercept in the linear region showed that the presence of covalent bonds at the interface resulted in a 55% increase in the first modulus and a 50% increase in the second modulus (Figure 4C). The scaffold with covalent bonds at the interface displayed higher stiffness and an approximate 60% increase in strain at break compared with the scaffold without covalent bonds (Figure 4C). It is evident that in the absence of covalent linkages between the hydrogel and the polymeric fibers, the mechanical properties of the fibers become dominant. Consequently, the hydrogel tends to seep out of the scaffold pores during tension (Figure 4A_3_), resulting in an overall more elastic scaffold. On the other hand, when the polymer and hydrogel are covalently linked, the hydrogel is less prone to breaking but instead follows the deformation process of the fibers (Figure 4B_4_). However, some resistance from the hydrogel is observed, contributing to lower strength and making the scaffold more brittle and less ductile overall.

Furthermore, it is noteworthy that the presence or absence of bonds at the interface between hydrogels and fibers imparts significant versatility to these scaffolds. Depending on the specific application requirements, such as the need for a softer yet elastic scaffold (like pHMGCL:PCL-silkMA) or a stronger but brittle scaffold (like pMHMGCL:PCL-silkMA), the incorporation or omission of interactions at the interface between the two scaffold components can be carefully considered. Combining the hydrogel with the support scaffold (with or without interfacial interactions) allows the freedom of tuning the mechanical properties, which is generally more complicated when using a hydrogel alone.

To further investigate the influence of the hydrogel and the interface interaction between the hydrogel and fibers, mechanical tensile tests were performed on scaffolds without silkMA hydrogel (Figure S6). The hydrogel-filled scaffolds exhibited an approximately 2-fold increase in stress at the breaking point compared to individual thermoplastic polymer scaffolds. Additionally, there was an approximately 2-fold increase in the tensile modulus with the pMHMGCL:PCL scaffold cross-linked with the silkMA hydrogel in comparison to the same scaffold without hydrogel. These findings indicate that the presence of the hydrogel soaked in the fibers enhances the mechanical performance, which is further augmented by the interfacial interactions between thermoplastic fibers and hydrogel.

In conclusion, mechanical testing revealed that the incorporation of covalent bonds at the hydrogel–fiber interface significantly enhanced the mechanical properties of the composite, resulting in increased stiffness and brittleness.

### 3.5 Biological Compatibility and Functionality of Polymer Blends

**Figure 5:**
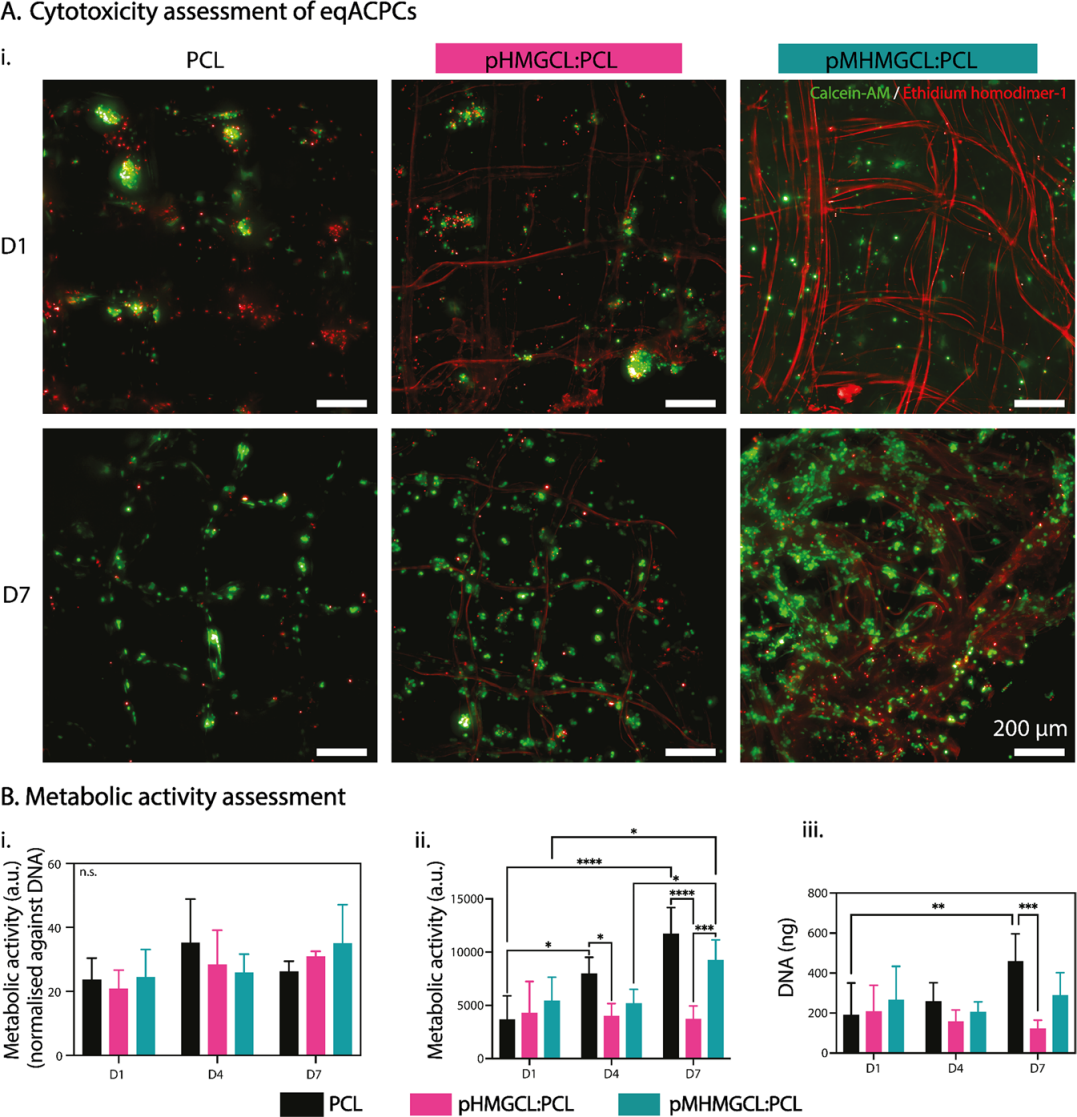
Cell-viability assessment of eqACPCs cultured directly on MEW scaffolds in expansion medium. (A) Live (green)/dead (red) viability assay maximum projection fluorescent microscopy images on D1 and D7. N.B. Background staining of MEW fibers is observable in these images, likely due to the physisorption of the less hydrophobic surfaces. *n* = 2; scale bar = 200 μm. (B) Metabolic activity assessment of constructs on D1, D4, and D7. *n* = 4, data shown as the mean ± standard deviation. Significance values: n.s. *p* > 0.05, *p ≤ 0.05, **p ≤ 0.01, ***p ≤ 0.001, ****p ≤ 0.0001. (i) Metabolic activity quantification normalized against DNA content of sample. Separate assay results are displayed as ii. Metabolic assay, and as iii. DNA quantification.

To assess the cytocompatibility of the modified polymer blends, ACPCs were expanded directly on MEW meshes made from the two blends (without hydrogel) for 7 days. Live/dead imaging showed clusters of living cells and small clusters of dead cells on D1 in all groups (Figure 5A). The prevalence of live cells in comparison to dead cells was consistent across the control (PCL) and blended groups (pHMGCL:PCL and pMHMGCL:PCL). There were no noticeable differences in the observable proportions of live to dead cells across the different groups after 7 days of *in vitro* culture (Figure 5A). D7 live/dead imaging revealed an increase in proliferation across the scaffold fibers (Figure 5A). Some instances of cells bridging the pore corners were observed in the pHMGCL:PCL and pMHMGCL:PCL blend scaffolds (Figure 5A). This could be related to the smaller fiber diameters (∼3 μm), and thus bigger surface areas, of the polymer blends in comparison to the PCL (∼10 μm), further enabling cellular attachment and therefore expediting proliferation and thus bridging. It was previously hypothesized that when cells are attaching to a fiber close to its size, then the cell is forced to adhere to the surface of the fiber, while when the fiber has a smaller diameter, it may be possible for the cell to wrap the fiber and thus more likely to bridge between two small fibers.^66^ The fiber diameter is known to affect cell attachment, morphology, and alignment, as well as guided differentiation in various cell types.^69−71^ Further investigations into morphology of ACPCs dependent on fiber diameter, particularly in the resolution range of MEW, would need to be performed to assess this. When the metabolic activity is normalized against DNA content (Figure 5Bi), no significant differences between any scaffold group nor time point of culture were observed. This is indicative that the increase in metabolic activity is correlated to the cell number increase. It may be noted that both the pHMGCL:PCL and pMHMGCL:PCL blend groups independently present an upward trend of normalized metabolic activity as the culture period progresses although not statistically significant. When analyzed without DNA normalization, the greatest metabolic activity increase was observed in the PCL group, with an increase in recorded activity between each time point. The pMHMGCL:PCL blend group also exhibited enhanced metabolic activity at the D7 time point, in comparison to the D1 and D4 readouts (∼2-fold). pHMGCL:PCL, on the other hand, maintained a consistent metabolic activity level throughout the culture period (Figure 5Bii). Previous metabolic assays (WST-1) on pHMGCL:PCL FDM-printed scaffolds also showed a significant increase in metabolic activity over time,^54^ aligning with the results of the pMHMGCL:PCL blend group, but would be contingent on the additional 4 days in culture. However, the pHMGCL:PCL group did not follow that trajectory based on the results from 7 days in culture. This difference may be due to the significant difference in scaffold design, particularly the fiber diameter achieved by MEW. DNA quantification alone showed a >2-fold increase in DNA content in the PCL control group over the 7-day culture period, whereas there were no significant changes observed in the pHMGCL:PCL or pMHMGCL:PCL blend groups across the time points (Figure 5Biii).

**Figure 6:**
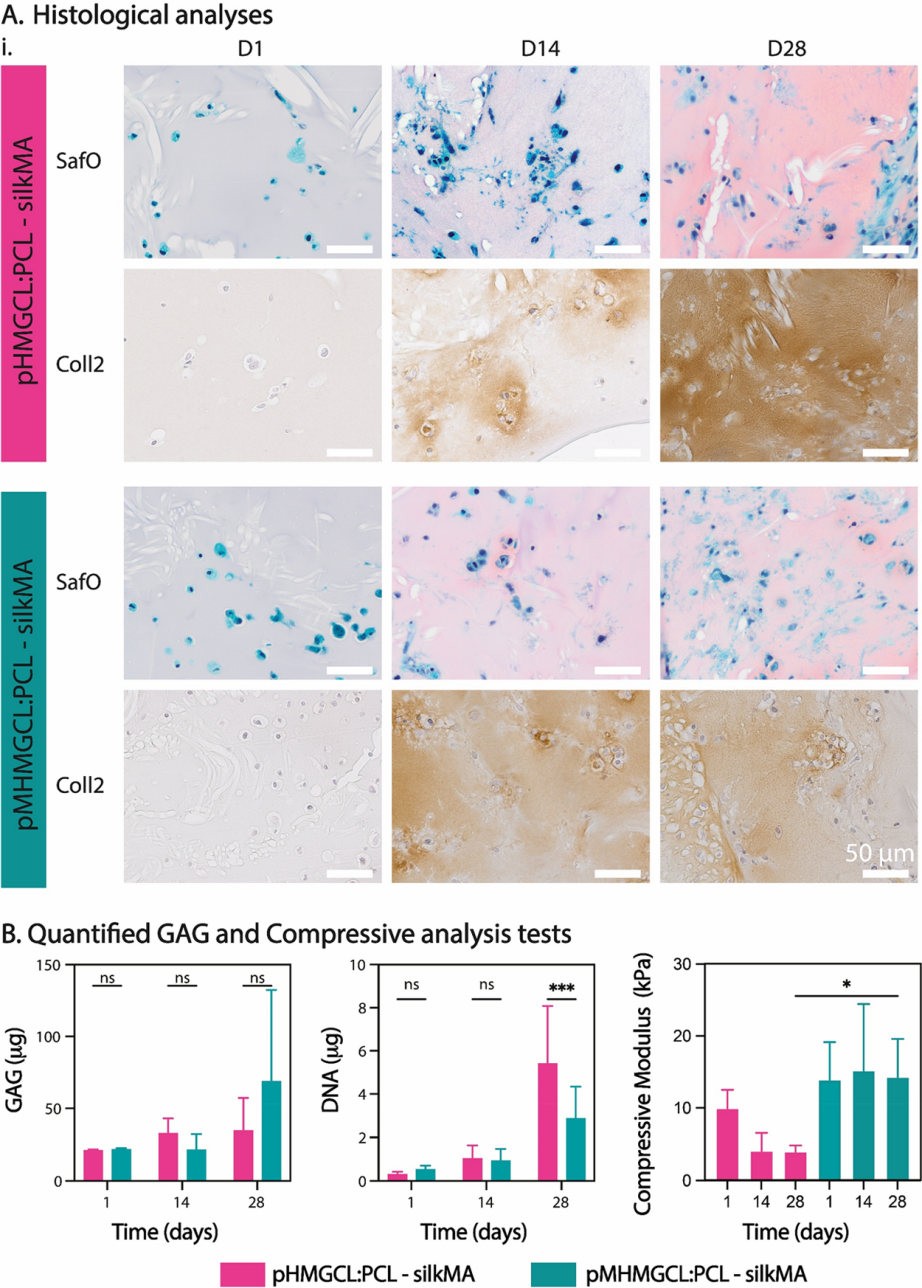
Chondrogenic differentiation assessment of eqACPCs cultured in pHMGCL:PCL or pMHMGCL:PCL MEW constructs, reinforced with SilkMA. Samples were cultured in chondrogenic differentiation medium. All analyses performed on samples taken from culture at D1, D14, and D28. (A) Histological analyses of paraffin-embedded samples. The top panel of each treatment group shows Saf-O/fast green (pink = GAGs), and the bottom panel shows collagen type II immunohistochemistry (brown = collagen II). Representative image shown. *n* = 3. Scale bar = 50 μm. (B) (i) Quantified glycosaminoglycans (GAGs) of digested samples (total GAGs/construct). Data shown as mean ± standard deviation. *n* = 5. (ii) DNA content of digested samples. Data shown as mean ± standard deviation. *n* = 5. Significance values: n.s. *p* > 0.05, *p ≤ 0.05, **p ≤ 0.01, ***p ≤ 0.001, ****p ≤ 0.0001. (iii) Compressive analysis of samples prior to digestion. Data shown as mean ± standard deviation. *n* = 5.

Given that the pHMGCL:PCL and pMHMGCL:PCL blends did not differ in cell viability compared to PCL *in vitro*, subsequent analysis focuses on examining the differences using methacrylate-mediated covalent attachment within a mesh-reinforced hydrogel culture model. Chondrogenic differentiation of eqACPCs embedded within the silkMA hydrogel was performed for a 28-day culture period to assess the biological functional implications of the observed differences in the compression modulus (Figure 6B) of the pHMGCL:PCL and pMHMGCL:PCL MEW meshes. Histological analysis of native-like articular cartilage matrix components, proteoglycans, and collagen type II demonstrated positive staining and gradual increased deposition along the culture period (Figure 6A). This indicated that chondrogenic differentiation and subsequent cartilage-like matrix deposition were achieved in both scaffold groups. A higher degree of deposited matrix homogeneity could be seen in the pMHMGCL:PCL group by D14, with the pHMGCL:PCL group demonstrating a positive detection of matrix proteins, albeit with a heterogeneous distribution, particularly when collagen type II deposition was examined (Figure 6A). At D28, both scaffold groups display qualitatively comparable levels of positively stained proteoglycans and collagen type II (Figure 6A). Collagen type I and collagen type VI deposition was also observed in both groups at D28, but no observable differences between the groups were seen (Figure S7. Collagen type I was observed around the edges of the constructs, but to a lesser extent than collagen type II (Figure S7), which is typical for the articular cartilage phenotype. Collagen type VI was detected around cells scattered throughout the constructs (Figure S7), which aligns with past studies that found that collagen type VI was important for the mechanical properties of the pericellular matrix.^72^

To quantify the proteoglycan matrix production, DMMB assay analyses exhibited continuous upward trend of increased glycosaminoglycan (GAG) production during the culture period in both groups, with a marked increase (>3-fold) in the pMHMGCL:PCL blend group at D28 reaching a 68.9 μg mean value, compared to samples from both D1 and D14, although not statistically significant due to high variance (Figure 6Bi). The pHMGCL:PCL blend group exhibited a 1.5-fold increase from 21.1 to 32.8 μg from D1 to D14, which plateaued to D28 at 34.8 μg. Cell proliferation was also quantified by measuring DNA content of the samples and was found to show a continuous increase throughout the culture period, with the largest increase seen between D14 and D28 (Figure 6Bii). This trend was most amplified in the pHMGCL:PCL scaffold group at D28, with an ∼3-fold increase between D14 and D28, while the pMHMGCL:PCL scaffold group revealed an ∼2-fold increase in the same period. When comparing between the two scaffold groups at D28, there is a statistical significance (*p* = 0.0006) in the increase of DNA content measured in the pHMGCL:PCL group (5.4 μg) compared to the pMHMGCL:PCL group (2.9 μg), despite the observed variability (Figure 6Bii). When normalizing the GAGs produced against DNA content at D28, the pHMGCL:PCL group exhibited 5.6 ± 2.3 μg GAG/μg DNA, while the pMHMGCL:PCL exhibited 20.8 ± 10.4 μg GAG/μg DNA, both in the range of previous studies using chondrocytes with the same polymer blends in a gelatin methacrylate-reinforced construct.^55^

Samples were also tested for their resistance against compressive forces throughout the culture period (end point analysis). Analyses demonstrate that the pMHMGCL:PCL group maintains a consistent compressive modulus throughout the culture period, whereas pHMGCL:PCL exhibits a reduction in bulk strength over time *in vitro*.

This may be attributable to a slight swelling of the silkMA hydrogel over a 28-day period, which may be limited in pMHMGCL:PCL-silkMA scaffolds due to the presence of covalent bonds between hydrogel with the fibers.^73,74^ Furthermore, the tests showed that after 28 days an approximately 3-fold difference in compression modulus value was observed between samples with and without covalent interactions at the interface between the materials.

Moreover, as shown in Figure S8, a significant increase in compression modulus is observed in the pMHMGCL-silkMA samples with eqACPCs compared to the cell-free controls, which is in contrast to the samples without the interface interactions (pHMGCL-silkMA) where no significant difference in compression modulus is observed for samples with and without embedded cells.

In summary, cell viability assessments demonstrated that the modified polymer blends (pHMGCL:PCL and pMHMGCL:PCL) supported cell growth and exhibited similar metabolic activity compared to the control (PCL) *in vitro*. Chondrogenic differentiation experiments confirmed the successful production of cartilage-like matrix in both scaffold groups, with marginally more homogeneous matrix deposition observed in the pMHMGCL:PCL blend scaffolds at early time points. Moreover, the pMHMGCL:PCL blend scaffolds exhibited consistent compressive strength throughout the culture period, whereas the pHMGCL:PCL scaffolds showed a reduction in bulk strength over time.

## 4 Conclusions

Overall, these findings highlight that the addition of a support structure to the hydrogel can provide the freedom to tune the mechanical properties of a hydrogel, sometimes unachievable in simple hydrogel formulations. In addition, covalent interactions at the interface between hydrogels and reinforcement scaffolds have a considerable influence on mechanical properties and cell behavior in composite scaffolds.

The incorporation of pMHMGCL:PCL melt electrowritten reinforcing scaffolds into silkMA hydrogels demonstrated improved mechanical properties and supported chondrogenic differentiation, showcasing their potential for tissue engineering applications requiring enhanced mechanical strength and functional tissue formation. The study underscores the significance of tailored scaffold designs to optimize interface interactions and meet specific tissue engineering requirements.

## Supporting Information Available

(1) Nuclear magnetic resonance spectroscopy of silkMA and pBMGCL, degree of modification for pMHMGCL; (2) thermogravimetric analysis of pHMGCL:PCL and pMHMGCL:PCL; (3) swelling and degradation of silkMA hydrogel; (4) stress–strain curves for uniaxial tensile tests of pHMGCL:PCL-silkMA and pMHMGCL:PCL-silkMA and stress–strain curves of pHMGCL:PCL and pMHMGCL:PCL scaffolds without hydrogel; (5) histological analysis of samples at D28 *in vitro*; (6) compressive analysis testing of pHMGCL:PCL-silkMA and pMHMGCL:PCL-silkMA samples prior to digestion showing compressive modulus (PDF)

## Abbreviations

BMG: benzyloxymethyl glycolide
CD: collector distance
CL: ε-caprolactone
CS: collector speed
CTS: critical translation speed
DNA: deoxyribonucleic acid
DSC: differential scanning calorimetry
GAG: glycosaminoglycan
GPC: gel permeation chromatography
LAP: lithium phenyl 2,4,6-trimethylbenzoylphosphinate
MEW: melt electrowriting
MR: magnetic resonance
*p*: pressure
pBMGCL: (poly(benzyloxymethyl glycolide-*co*-ε-caprolactone)
PBS: phosphate buffered saline
pHMGCL: poly(hydroxymethylglycolide-*co*-ε-caprolactone)
pMHMGCL: poly(hydroxymethylglycolide-*co*-ε-caprolactone) functionalized with methacrylic groups
Saf-O: Safranin-O
SF: silk fibroin
silkMA: silk fibroin methacryloyl
TGA: thermogravimetric analysis
*V*: voltage
