# Supplementary material for: Covalent Grafting of Functionalized MEW Fibers to Silk Fibroin Hydrogels to Obtain Reinforced Tissue Engineered Constructs
Source: Biomacromolecules. 2024 Feb 7;25(3):1563–77. doi: 10.1021/acs.biomac.3c01147 (PMC10934835; doi:10.1021/acs.biomac.3c01147)
Supplement: Supplementary file 1 — bm3c01147_si_001.pdf [file bm3c01147_si_001.pdf]

SUPPORTING INFORMATION

Covalent grafting of functionalized MEW fibers to silk fibroin hydrogels to obtain reinforced tissue engineered constructs

Martina Viola, Madison J. Ainsworth, Marko Mihajlovic, Gerardo Cedillo-Servin, Mies J. van Steenbergen, Mattie van Rijen, Mylène de Ruijter, Miguel Castilho, Jos Malda, Tina Vermonden

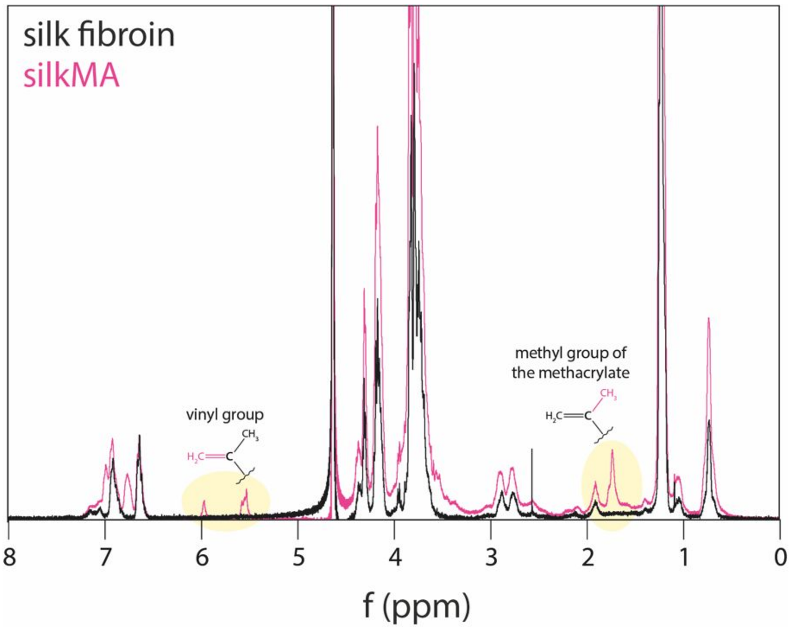

Supplemental figure 1. ^{1}H-NMR analysis of silkMA

A. pBMGCL ^{1}HNMR

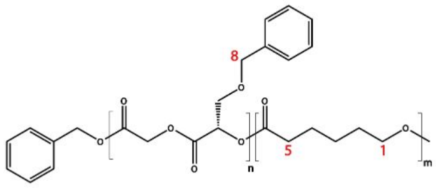

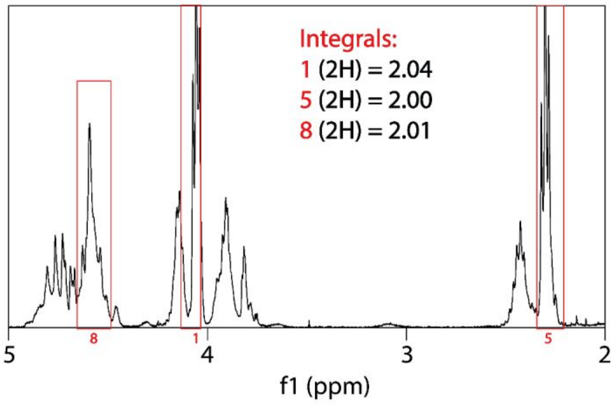

From this NMR we take as a reference the signals:
1CH_2 (4.0-4.1 ppm) and 5CH_2 (2.2-2.3 ppm).
The integral of both these signals should be = 2
As a reference for the BMG we use the signal at 4.5 which correspond to 8CH_2
The integral of that is also = 2, meaning that the ratio between BMG and CL is 1:1 instead of 3:2.

B. Degree of modification

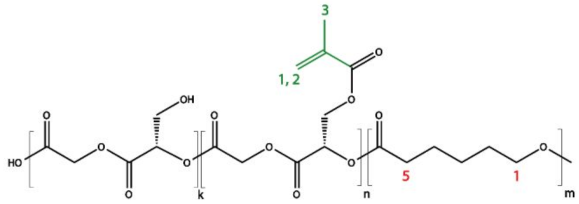

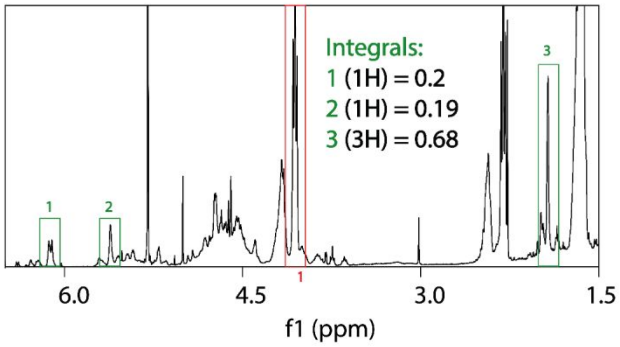

We use as reference peak always 1CH_2 (4.0-4.1 ppm) which integrated is = 2. The signals of methacrylic anhydride are at 1.9 ppm, 5.6 ppm and 6.1 ppm. Since the integration of both peaks at 5.6 and 6.1 are around 0.2 and the one at 1.9 is 3 times higher we can conclude that we have almost 20% DoM in the BMG part of the polymer.

Supplemental figure 2. NMR analysis A. BMG:CL ratio calculation; B. Degree of modification

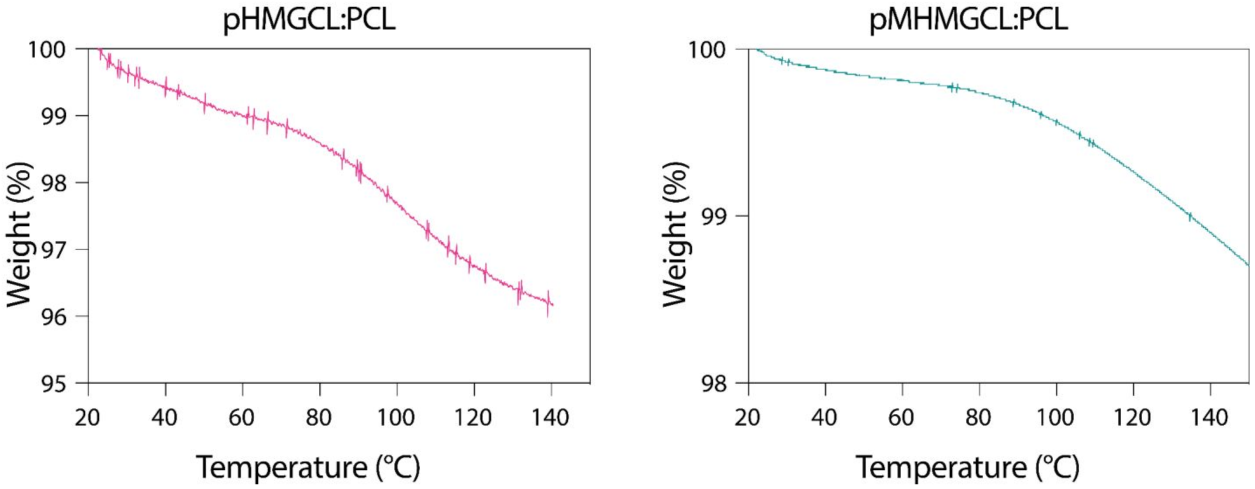

Supplemental figure 3. TGA analysis: degradation of pHMGCL:PCL and pMHMGCL:PCL polymer blends from RT to 140°C

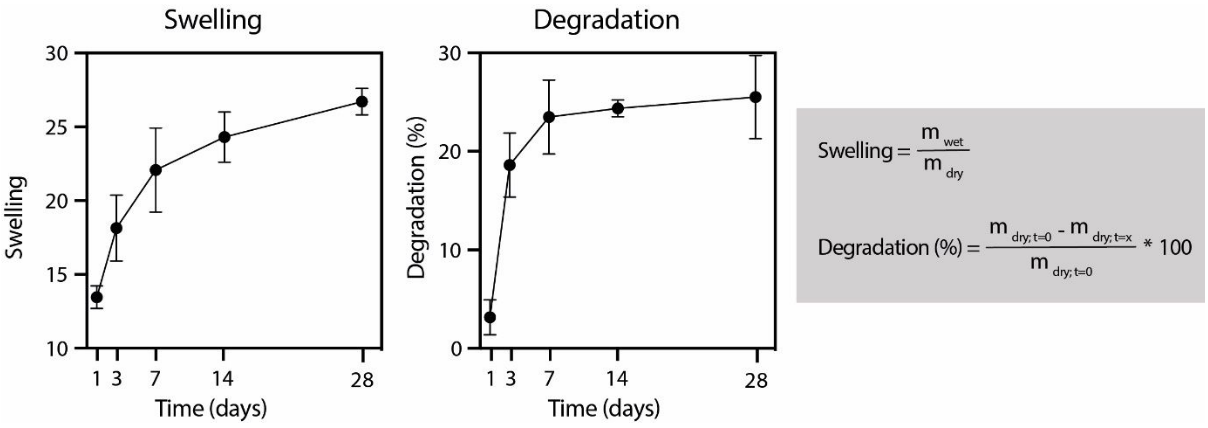

Supplemental figure 4. Swelling and degradation of a silkMA (7 % w v^{-1}) hydrogel with LAP (0.1 w v^{-1}) over 28 days (n = 3).

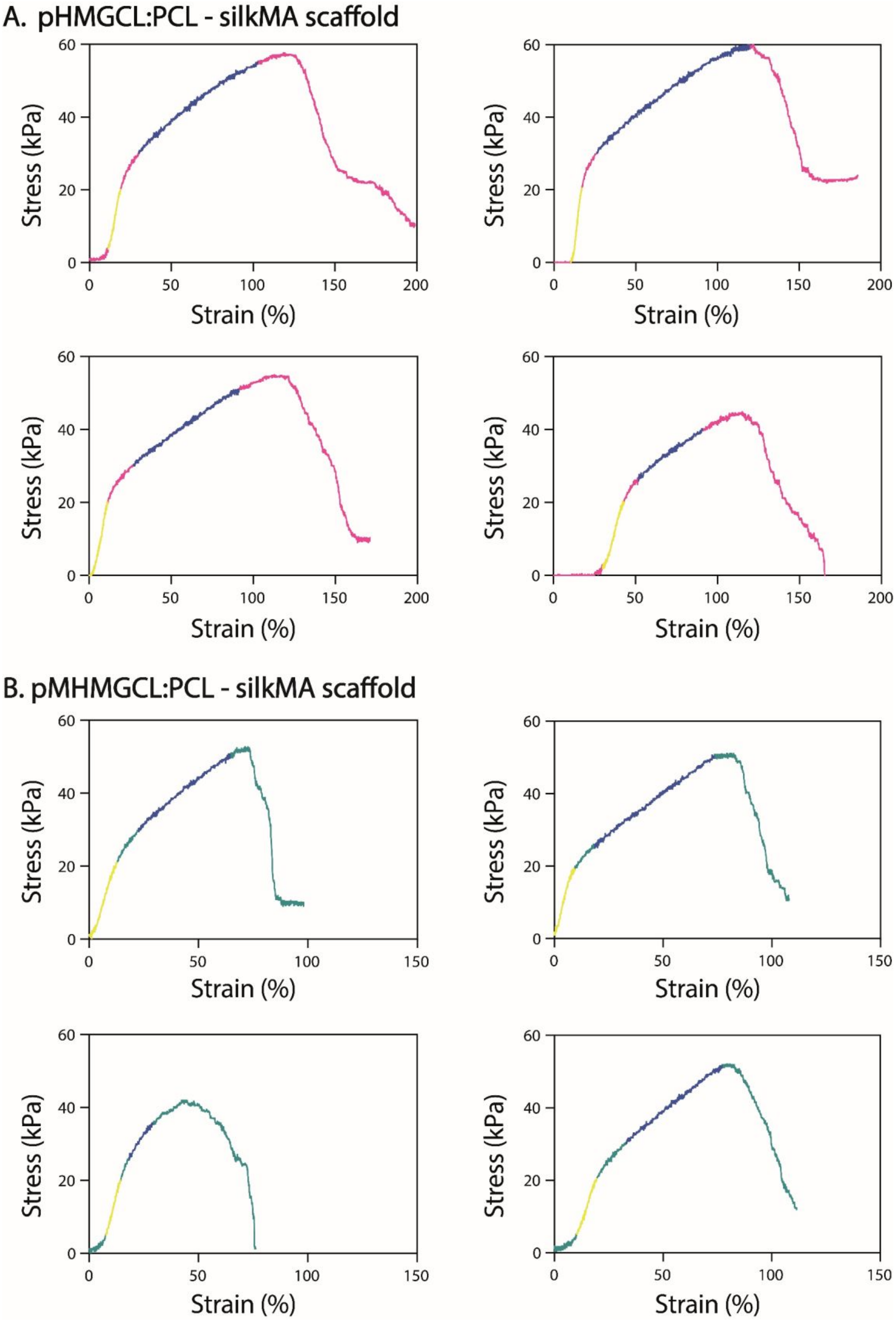

Supplemental figure 5. Mechanical characterization: curves for the uniaxial tensile tests of A. pHMGCL:PCL-silkMA (pink) and B. pMHMGCL:PCL-silkMA (green) scaffolds.

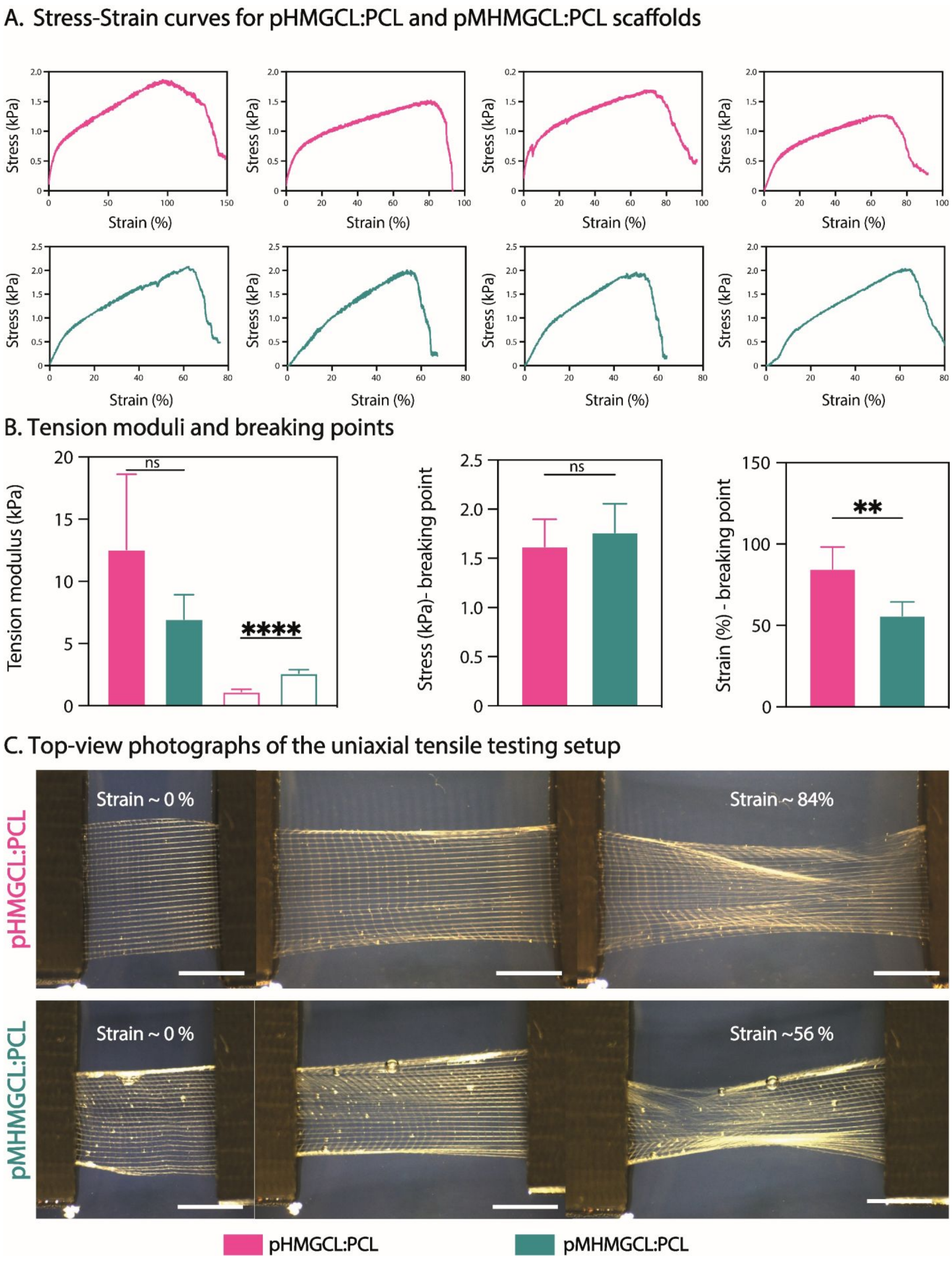

Supplemental figure 6. Mechanical characterization: A. curves for the uniaxial tensile tests of pHMGCL:PCL (pink) and pMHMGCL:PCL (green) scaffolds. B. Tensile modulus calculated using the

first and second slope of the curves, values of the stress and the strain at the breaking point for pHMGCL:PCL and pMHMGCL:PCL scaffolds (n=4), significance values: $^{**}p \leq 0.01$, $^{****}p \leq 0.0001$. C. top-view photographs of the uniaxial tensile testing setup for pHMGCL:PCL and pMHMGCL:PCL: starting point, maximum elastic elongation and breaking point. Scale bar = 5 mm.

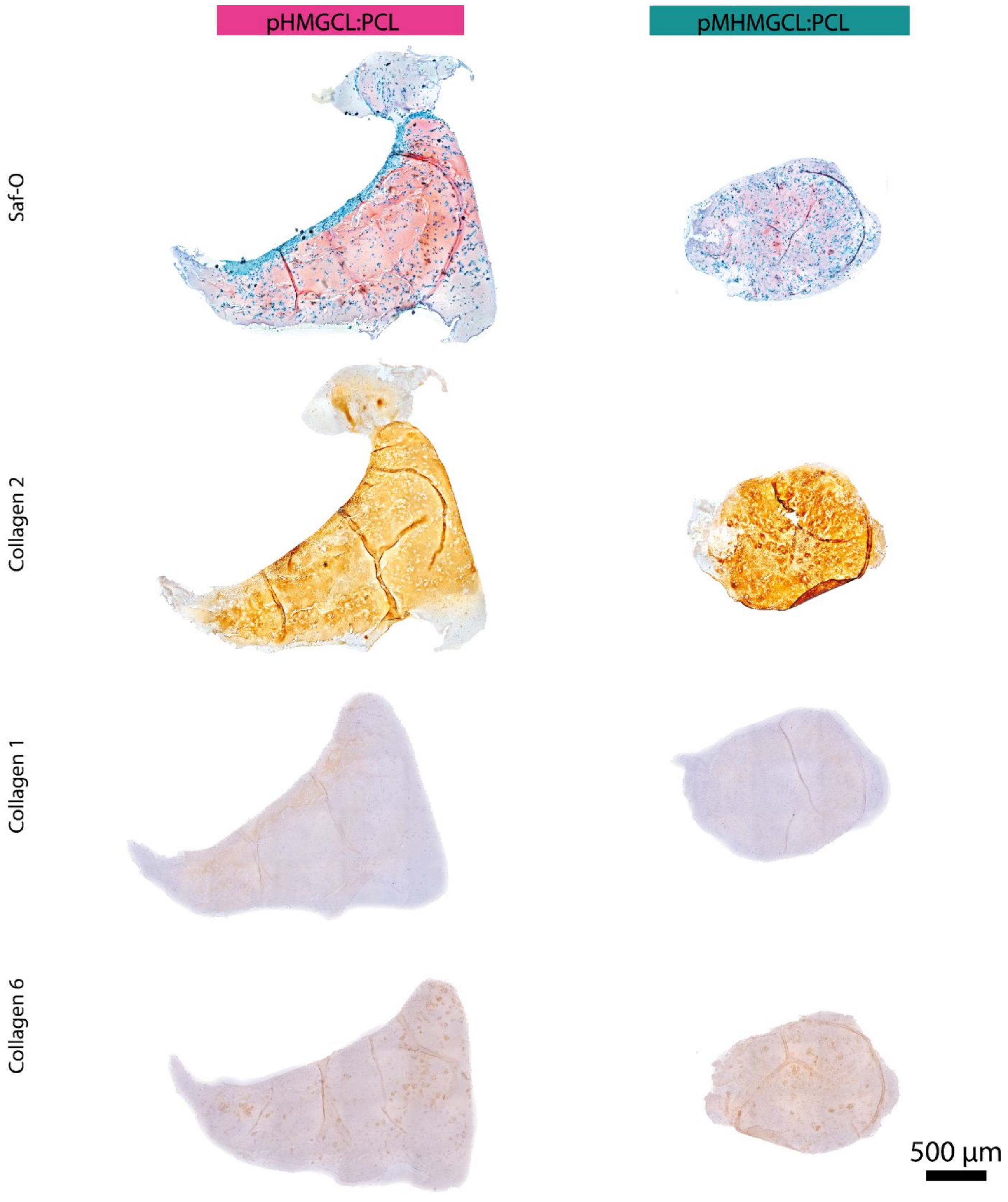

Supplemental figure 7. Overview (tile scans of whole samples) of histological analysis of samples at D28 *in vitro*. Representative image shown. n = 3. Scale bar = 500 µm.

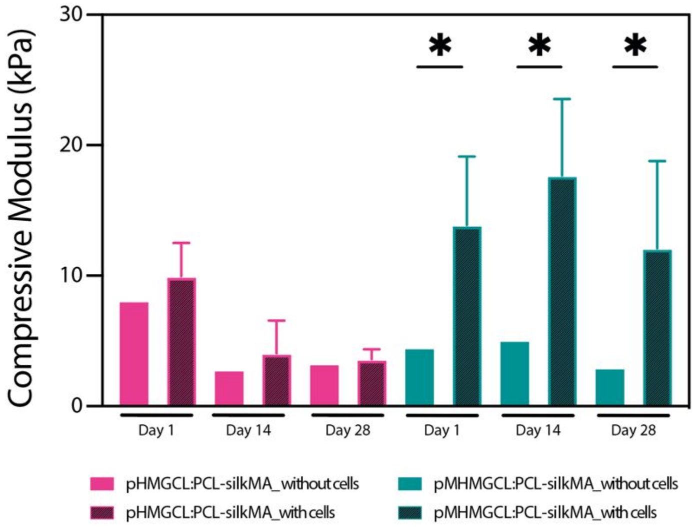

Supplementary figure 8. Compressive analysis testing of samples prior to digestion showing compressive modulus. Data shown as mean ± standard deviation (n = 5). The statistics show non-significant differences between samples unless indicated otherwise.
